# FirePM-YOLO: Position-Enhanced Mamba for YOLO-Based Fire Rescue Object Detection from UAV Perspectives

**DOI:** 10.3390/s26072064

**Published:** 2026-03-26

**Authors:** Qingyu Xu, Runtong Zhang, Zihuan Qiu, Fanman Meng

**Affiliations:** School of Information and Communication Enginnering, University of Electronic Science and Technology of China, Chengdu 611731, China; 202312012432@std.uestc.edu.cn (Q.X.); rtzhang@std.uestc.edu.cn (R.Z.); zihuanqiu@std.uestc.edu.cn (Z.Q.)

**Keywords:** object detection, selective state space, fire rescue, unmanned aerial vehicle reconnaissance

## Abstract

Object detection in UAV-based fire rescue scenarios faces multiple challenges, including densely distributed small targets, severe occlusion, and interference from smoke and flames. Existing mainstream detection models, such as the YOLO series, often prioritize inference speed at the expense of modeling global context and spatial positional information, resulting in limited performance in such complex environments. To address these limitations, this paper proposes FirePM-YOLO, an object detection architecture optimized for fire rescue applications. Based on the YOLO framework, the proposed model introduces two key innovations: first, a Position-Aware Enhanced Mamba module (PEMamba) is designed, which incorporates a compact positional encoding mechanism, lightweight spatial enhancement, and an adaptive feature fusion strategy to significantly improve scene perception while maintaining computational efficiency. Second, a PEMBottleneck structure is constructed, which dynamically balances local convolutional features and global PEMamba features via learnable weights. This module is embedded into the shallow layers of the backbone network, forming an enhanced PEM-C3K2 module that captures long-range dependencies with linear complexity while preserving fine local details, thereby enabling holistic contextual understanding of fireground environments. Experimental results on the self-built “FireRescue” dataset demonstrate that compared with the original YOLOv12 and other mainstream detectors, the proposed model achieves improvements in both mean average precision (mAP) and recall while maintaining real-time inference capability. Notably, it exhibits superior detection performance on challenging samples, such as small-scale and partially occluded professional firefighting vehicles.

## 1. Introduction

With the increasing frequency of natural disasters and accidents worldwide, UAV-based object detection for fire rescue scenarios has become a critical component of modern fire rescue command systems [1]. However, the inherent complexity and unique challenges of these scenarios impose severe demands on existing detection methods. First, UAVs typically adopt a high-altitude, wide-area reconnaissance perspective, resulting in an extremely low pixel proportion of critical targets (e.g., fire trucks and firefighters) within images, constituting a classic small-object detection problem. Traditional methods often suffer from missed detections due to insufficient feature information. Second, the on-scene environment presents extreme visual interference, where dense smoke, flames, water mist, dust, and structural debris severely degrade image quality, manifesting as blurring, reduced contrast, and feature occlusion. This places stricter requirements on the robustness of feature extraction under such extreme conditions. Additionally, target similarity and severe occlusion further exacerbate detection difficulty: specialized rescue vehicles closely resemble ordinary civilian trucks in both color and shape, while dense deployment or complex terrain often leads to partial or complete occlusion of targets, easily causing model confusion and cascading false detections. Consequently, algorithms must be capable of accurately distinguishing similar objects in cluttered environments.

**Figure 1:**
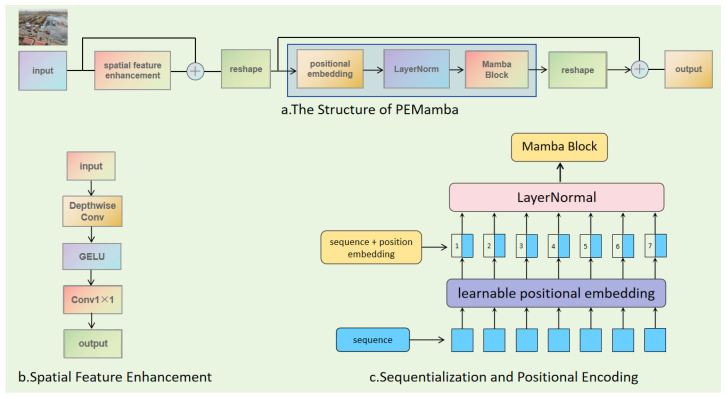
Overall architecture of PEMamba. (**a**) The structure of PEMamba. This section illustrates the end-to-end framework of the proposed PEMamba model, detailing the flow of input data through its core components. (**b**) Spatial feature enhancement. This subfigure demonstrates the mechanism for enhancing spatial features, highlighting how the model emphasizes critical spatial information within the feature maps. (**c**) Sequentialization and positional encoding. This part depicts the process of transforming 2D feature maps into 1D sequential tokens and the subsequent injection of positional information to retain spatial structure awareness.

The YOLO (You Only Look Once) series is selected as the foundational architecture based on the following considerations. First, YOLO models are renowned for their exceptional balance between detection accuracy and inference speed, establishing them as the de facto standard for real-time object detection tasks. Given that UAV-based fire rescue scenarios demand immediate and efficient processing to support time-critical decision-making, the real-time capability of YOLO is indispensable. Second, the YOLO framework exhibits strong modularity and flexibility, which facilitates the integration of novel components such as the proposed PEMamba module (Position-Aware Enhanced Mamba; see Figure 1a) without disrupting the overall architecture. Third, the recently released YOLOv12 [2] introduces attention-centric improvements that enhance feature representation while maintaining efficiency, providing a solid and up-to-date baseline for further innovation.

Although YOLOv12 excels in general object detection tasks, its design, which is primarily intended for conventional scenarios, introduces inherent limitations when applied to the specific domain of fire rescue. Specifically, fire rescue scenarios present challenges such as complex spatial dependencies, densely distributed small objects, and severe occlusion. However, the backbone network of YOLOv12 still predominantly relies on convolutional operations, lacking effective modeling of global contextual relationships and spatial position awareness. These limitations manifest in three main aspects: a restricted local receptive field that hinders global information capture, insufficient contextual modeling for small objects, and inadequate multi-scale feature fusion mechanisms. Consequently, when processing fire rescue scene images, YOLOv12 struggles to adequately perceive critical targets in complex environments. Therefore, this paper selects YOLOv12 as the baseline model to preserve its real-time detection advantages and attention-enhanced mechanisms while systematically addressing its aforementioned limitations in fire rescue scenarios. By introducing the PEMamba module to enhance global contextual modeling and position awareness, we not only compensate for the shortcomings of the original model but also demonstrate the effectiveness and generalizability of our proposed improvements.

Selective state space models (SSMs), exemplified by Mamba [3], have recently demonstrated remarkable advantages in long-sequence modeling tasks. Their linear computational complexity and favorable scalability position them as strong contenders to Transformers. However, when applying Mamba to computer vision tasks, particularly small object detection, several critical challenges emerge: (1) loss of spatial structural information, as the native 1D sequential design of Mamba necessitates flattening 2D feature maps, thereby disrupting inherent spatial adjacency relations; (2) weakened features of small objects, whose limited pixel coverage leads to feature responses that are easily overwhelmed by larger objects and background noise; (3) insufficient multi-scale adaptability, where fixed-scale positional encodings struggle to meet the localization accuracy requirements for objects of varying sizes; (4) disconnection between local and global information, as Mamba’s global sequence processing may overlook the synergistic relationship between local details and global semantics. VMamba [4] and Vision Mamba [5] have successfully introduced the Mamba architecture into vision tasks, achieving linear computational complexity while maintaining a global receptive field. However, such methods still exhibit notable shortcomings in preserving local details, modeling spatial structures, and achieving practical computational efficiency—particularly in tasks such as small object detection, which demands highly fine-grained local perception. In our experiments, we observed that directly applying modules like Vision Mamba to mainstream detection models such as YOLOv12 still results in high computational complexity, prolonged training time, and low inference efficiency, making it difficult to meet the requirements of real-time scenarios like firefighting and rescue operations. These limitations suggest that pure Vision Mamba architectures may underperform compared to carefully designed CNN-based or hybrid architectures in certain vision tasks.

To systematically address the unique challenges of small object detection in fire rescue scenarios, this paper proposes an innovative dual-path enhancement strategy. Rather than being a simple combination of existing techniques, this strategy delves into the essence of the problem to deeply optimize the feature extraction paradigm. First, we identify a core defect of the Mamba architecture in vision tasks—the positional perception blind spot—and design the Position-Aware Enhanced Mamba (PEMamba) module to resolve it. Second, we overcome the challenge of efficiently integrating state space models with convolutional detection frameworks by seamlessly incorporating PEMamba into YOLOv12, thereby constructing the FirePM-YOLO detection system. Specifically, the main contributions of this paper are summarized as follows:

(1) We uncover the positional perception deficiency of Mamba in visual object detection and propose the PEMamba module to address it. The scanning process of the original Mamba’s state space model inherently collapses two-dimensional spatial structures into one-dimensional sequences when processing visual signals, leading to the critical loss of spatial position information. To tackle this fundamental issue, we introduce the PEMamba module. Rather than simply appending positional encoding, this module explicitly reconstructs two-dimensional spatial awareness within the state space model through the synergistic integration of three components: a compact positional encoding mechanism, a lightweight spatial enhancement scheme, and an adaptive feature fusion strategy. It significantly enhances the model’s global perception and positional awareness in complex fire environments while preserving Mamba’s advantage of linear complexity, thereby providing a theoretical foundation for addressing the challenge of inaccurate small object localization.
(2) We propose the PEMBottleneck structure and the enhanced PEM-C3K2 module to address the challenge of efficient and dynamic fusion between global and local features. Directly inserting PEMamba into the YOLO framework leads to feature conflicts and computational redundancy. To mitigate this, we design the PEMBottleneck structure, which employs a learnable gating mechanism to dynamically fuse local convolutional features (excelling at detail extraction) with global state space features extracted by PEMamba (specializing in long-range dependency modeling). The resulting PEM-C3K2 module enables fine-grained feature preservation for occluded and small-scale targets while modeling long-range dependencies with linear computational complexity, fundamentally enhancing the model’s discriminative capability in complex scenarios.
(3) We construct FirePM-YOLO, an efficient and robust detection framework tailored for fire rescue scenarios. This is not a mere application of an existing model but a system-level optimization solution. By leveraging the real-time advantages of YOLOv12 and integrating the two aforementioned levels of innovation, FirePM-YOLO systematically tackles three core challenges in fire rescue: dense small objects, severe occlusion, and smoke interference. The framework achieves significantly improved detection accuracy while maintaining real-time inference efficiency, offering a novel technical pathway for fire scene reconnaissance.

Comprehensive experiments are conducted on our self-constructed FireRescue dataset [6]. The results demonstrate that FirePM-YOLO achieves substantial improvements in key metrics such as mean average precision (mAP) and recall compared to state-of-the-art detectors. Notably, our method exhibits exceptional robustness on challenging samples involving small objects, severe occlusion, and visual interference. The contribution of this research extends beyond proposing a novel detection method; it explores a new paradigm for the deep integration of state space models and convolutional neural networks in object detection tasks, offering a solution with both theoretical depth and practical value to the long-standing challenge of small object detection.

The remainder of this paper is organized as follows: Section 2 reviews related work. Section 3 elaborates on the overall architecture of FirePM-YOLO and its core improvement modules, with particular emphasis on the strategic placement of PEMamba in shallow layers—a design choice motivated by the need to preserve spatial resolution for small target detection while enabling early context integration—and the parallel fusion architecture in PEMBottleneck, which enables adaptive balancing of local details and global context. Section 4 introduces the experimental setup, dataset, and result analysis. Section 5 concludes the paper and outlines directions for future work.

## 2. Related Work

### 2.1. General Object Detection Algorithms

In recent years, computer vision has achieved significant advances in object detection, especially through deep learning-based algorithms [7], offering new possibilities for analyzing fire rescue scenes. Current deep learning-based object detection methods are broadly categorized into two-stage and one-stage approaches. Representative two-stage detectors such as R-CNN [8], Fast R-CNN [9], and Faster R-CNN [10] rely on pre-generated region proposals for object classification and localization, which generally require substantial computational resources and exhibit relatively slower inference speeds. In contrast, one-stage detectors like the YOLO series have established themselves as milestones in real-time object detection. Early models, including YOLOv1 [11], YOLOv2 [12], and YOLOv3 [13] laid the foundation for the series, with their performance gains closely tied to backbone enhancements and the widespread adoption of DarkNet. Subsequent versions introduced further innovations: YOLOv4 [14] incorporated CSPNet [15], YOLOv6 [16] adopted re-parameterization techniques, YOLOv7 [17] reorganized the network using ELAN, and YOLOv8 [18] introduced a decoupled head and anchor-free design. YOLOv9 [19] integrated attention mechanisms such as the Convolutional Block Attention Module (CBAM [20]) and Efficient Channel Attention (ECA [21]) into key backbone locations to improve focus on target regions. The more recent YOLOv10 [22] further incorporated transformer components by proposing the Partial Self-Attention (PSA) module, thereby enhancing global modeling capacity while maintaining manageable computational overhead. YOLOv11 [23] added a real-time attention visualization module and dynamically adjusted anchor sizes and ratios during training to better align with varying dataset distributions. Most recently, YOLOv12 [2] deeply integrates multiple attention mechanisms—including self-attention, CBAM, and ECA—across both its backbone and feature fusion layers, using a multi-branch design to strengthen feature representation without substantially increasing computational complexity. This ongoing evolution underscores the continued vitality and adaptability of the YOLO series in advancing real-time detection performance.

### 2.2. Object Detection in Disaster Management and Underwater Scenarios

Beyond general object detection methods, domain-specific detection tasks in disaster management and underwater environments share common challenges with fire rescue scenarios, such as small object scales, severe occlusions, and complex environmental interference.

In the field of disaster management, several studies have explored UAV-based detection techniques for post-disaster building detection and rescue, water rescue, and forest fire detection technology. For instance, Damjan et al. [24] employed lightweight-based detectors—RTMDet, YOLOv7, and YOLOv8—for building localization, followed by dedicated damage severity classification using state-of-the-art architectures, including Compact Convolutional Transformers, EfficientNet, and ResNet. Beigeng Zhao et al. [25] introduced a dataset rebalancing method based on a greedy algorithm to mitigate potential sample constraints by redistributing samples between training and validation sets. C. Yang et al. [26] introduced a novel feature fusion module called Trident Fusion, which is innovatively designed and incorporated into the neck of the model. Similar to fire rescue scenarios, these tasks also face challenges such as dynamic backgrounds and varying object scales.

Underwater object detection presents unique challenges, including low illumination, light absorption, and scattering effects, all of which lead to severe image degradation. Recent studies have addressed these issues through various improvements. Junlin Ouyang et al. [27] proposed an improved underwater object detection model based on YOLOv9, integrating advanced attention mechanisms and a dilated large-kernel algorithm. Yi et al. [28] introduced UWSC-YOLOv7, which incorporates the SENet attention mechanism and the EIoU loss function to improve detection accuracy without compromising speed. Pan et al. [29] introduced YOLOv10-AD, which adopts the AKVanillaNet backbone and dynamic snake convolution (DysnakeConv), combined with the PIoU loss function, to maintain high detection accuracy while significantly reducing parameter count.

Despite differences in application domains, disaster management and underwater detection share methodological commonalities with fire rescue detection: (1) the need for robust feature extraction under environmental interference; (2) challenges in detecting partially occluded targets; and (3) the demand for efficient architectures suitable for resource-constrained platforms. However, existing methods in these domains typically focus on their specific environmental degradation issues (e.g., underwater haze vs. fire smoke) and have not been systematically adapted for fire rescue scenarios. This gap motivates the development of a dedicated detection framework tailored to the unique characteristics of fireground environments.

### 2.3. YOLO-Based Attention Enhancement Methods

Many practitioners have enhanced these models by incorporating attention mechanisms, achieving desirable performance gains in their respective domains. For instance, Squeeze-and-Excitation (SENet) [30] block was integrated into an improved YOLACT [31] for identifying rumen protozoa in microscopic images. ViT-YOLO [32] integrates MHSA-Darknet into YOLO, supplemented with enhanced training strategies such as Test Time Augmentation (TTA) and weighted frame fusion. However, the increase in parameters and FLOPs did not yield the anticipated performance improvement, revealing the scalability limitations of Transformers in object detection tasks, particularly within the YOLO framework. Gold-YOLO [33] proposes extracting and fusing multi-scale feature information through a combination of convolutional and attentional primitives to enhance feature fusion. However, while these methods embed Transformer structures, they often sacrifice their core advantages—powerful global attention mechanisms and long-sequence processing capabilities. Concurrently, their attempts to mitigate computational explosion by reducing quadratic complexity frequently end up constraining model performance. Although Transformers excel at capturing long-range dependencies, the self-attention mechanism’s quadratic computational complexity grows with increasing input size, posing a significant challenge for high-resolution biomedical images due to the associated high computational cost.

### 2.4. Global Context Modeling Methods

Recently, methods based on state space models (SSMs), such as Mamba [3], have provided new ideas for solving these problems due to their strong modeling capabilities for long-distance dependencies and superior properties of linear time complexity. Recent advancements in state space models (SSMs) [34,35,36], particularly structured SSMs (S4) [37], offer a promising solution due to their efficient handling of long sequences. The Mamba model enhances S4 with selective mechanisms and hardware-aware optimizations, demonstrating superior performance in dense data domains [38]. The introduction of the Cross-Scan Module (CSM) in VMamba further extends its applicability to computer vision tasks by enabling spatial traversal and converting non-causal visual images into ordered patch sequences [4]. Vision Mamba [5] introduces the Mamba model into computer vision tasks, achieving linear computational complexity without sacrificing the global receptive field. EfficientVMamba [39] integrates an atrous-based selective scan approach by efficient skip sampling, constituting building blocks designed to harness both global and local representational features. MobileMamba [40] introduces the Multi-Receptive Field Feature Interaction (MRFFI) module—comprising the Long-Range Wavelet Transform-Enhanced Mamba (WTE-Mamba), Efficient Multi-Kernel Depthwise Deconvolution (MK-DeConv), and Redundant-Identity Elimination components—which balances efficiency and performance. Building upon this, Ref. [41] proposes the integration of Vision Mamba blocks (VSS) [5] within the U-Net architecture to enhance long-range dependency modeling for medical image analysis, resulting in Mamba-UNet. Mamba-YOLO [42] represents one of the earlier attempts to integrate the selective state space model (Mamba) with the YOLO architecture. This approach introduces Mamba modules into the backbone network of YOLO, aiming to model global contextual dependencies with linear complexity and mitigate the limitations of traditional convolutional operations in capturing long-range relationships. However, these works generally lack explicit spatial position awareness, fail to systematically establish a multi-scale feature fusion mechanism, and have not designed dedicated attention enhancement strategies to address specific challenges in complex scenarios, such as densely distributed small objects, severe occlusions, and inter-class similarity.

## 3. Methodology

This chapter is structured as follows: First, the foundational concepts and principles of state space models are introduced. Next, the design and implementation details of key enhanced modules are thoroughly analyzed and discussed. Finally, a high-level overview of the FirePM-YOLO architecture is provided.

### 3.1. Preliminaries

The structured state-space sequence models S4 [37] and Mamba [3], rooted in state space models (SSMs), both stem from a continuous system that maps a univariate sequence x(t)∈R into an output sequence y(t) via an implicit latent intermediate state $h\left(t\right)\in R^{N}$. This design not only bridges the relationship between inputs and outputs but also encapsulates temporal dynamics. The system can be mathematically defined as follows:

(1) $$h^{\prime }\left(t\right)=Ah\left(t\right)+Bx\left(t\right)$$

(2) y(t)=Ch(t)

In Equation (1), $A\in R^{N\times N}$ represents the state transition matrix, which governs how the hidden state evolves over time, while $B\in R^{N\times 1}$ denotes the weight matrix for the input space in relation to the hidden state. Moreover, $C\in R^{N\times 1}$ is the observation matrix, which maps the hidden intermediate state to the output. Mamba applies this continuous system to discrete-time sequence data by employing fixed discretization rules $f_{A}$ and $f_{B}$ to transform the parameters *A* and *B* into their discrete counterparts $\bar{A}$ and $\bar{B}$, respectively, thereby better integrating the system into deep learning architectures. A commonly used discretization method for this purpose is the Zero-Order Hold (ZOH). The discretized version can be defined as follows:

(3) $$\bar{A}=exp\left(\Delta A\right)$$

(4) $$\bar{B}={\left(\Delta A\right)}^{-1}\left(exp\left(\Delta A\right)-I\right)\Delta B$$

In Equations (3) and (4), Δ represents a time scale parameter that adjusts the temporal resolution of the model, and ΔA and ΔB correspondingly denote the discrete-time counterparts of the continuous parameters over the given time interval. Here, *I* represents the identity matrix. After transformation, the model is computed via linear recursive forms, which can be defined as follows:

(5) $$h^{\prime }\left(t\right)=\bar{A}h_{t-1}+\bar{B}x\left(t\right)$$

(6) $$y_{t}=Ch_{t}$$

The entire sequence transformation can also be represented in a convolutional form, which is defined as follows:

(7) $$\bar{K}=\left(C\bar{B},C\bar{AB},\ldots ,C{\bar{A}}^{L-1}\bar{B}\right)$$

(8) $$y=x*\bar{K}$$

where $\bar{K}\in R^{L}$ represents the structured convolutional kernel, with *L* denoting the length of the input sequence. In the design presented in this paper, the model employs a convolutional form for parallel training and utilizes a linear recursive formulation for efficient autoregressive inference.

### 3.2. PEMamba: Mamba-Based Vision Layer with Position-Aware Enhancement

We propose a novel Mamba-based vision layer with enhancement—Location-Aware Mamba (PEMamba)—which integrates a selective state space model with compact positional encoding operations to enable efficient visual feature modeling. Figure 1a illustrates the overall architecture of this layer, which consists of four core components: a lightweight spatial enhancement module, learnable positional embeddings, a Mamba state space module, and a learnable residual connection mechanism. The forward propagation process of PEMamba is outlined in Algorithm 1.
**Algorithm 1** Forward Computation Flow of PEMamba**Input feature map:** $X\in R^{B\times C\times H\times W}$**Output feature map:** $X_{\mathrm{out}}\in R^{B\times C\times H\times W}$**Parameters:** *B*: batch size, *C*: channels, *H*: height, *W*: width Epos← learnable position embedding; α← learnable residual weight (initialized as 0.1);$X\leftarrow X+0.1\cdot F_{\mathrm{spatial}}\left(X\right)$ //Lightweight spatial feature enhancementL←H×W //Sequence length$X_{\mathrm{seq}}\leftarrow \mathrm{reshape}\left(X,\left[B,C,L\right]\right)$ //Flatten spatial dimensions$X_{\mathrm{seq}}\leftarrow \mathrm{transpose}\left(X_{\mathrm{seq}},1,2\right)$ //[B,L,C]$X_{\mathrm{pos}}\leftarrow X_{\mathrm{seq}}+E_{\mathrm{pos}}$ //Add position encoding (broadcasting)$X_{\mathrm{norm}}\leftarrow \mathrm{LayerNorm}\left(X_{\mathrm{pos}}\right)$ //Layer normalization$X_{\mathrm{mamba}}\leftarrow \mathrm{Mamba}\left(X_{\mathrm{norm}}\right)$ //Mamba state space model$X_{\mathrm{mamba}}\leftarrow \mathrm{transpose}\left(X_{\mathrm{mamba}},1,2\right)$ //[B,C,L]$X_{\mathrm{mamba}}\leftarrow \mathrm{reshape}\left(X_{\mathrm{mamba}},\left[B,C,H,W\right]\right)$ //Restore spatial shape$X_{\mathrm{out}}\leftarrow X+\alpha \cdot X_{\mathrm{mamba}}$ //Adaptive residual connection**Return** $X_{\mathrm{out}}$

#### 3.2.1. Overall Architecture of PEMamba Module

Given an input feature tensor $X\in R^{B\times C\times H\times W}$, where *B*, *C*, *H*, and *W* denote batch size, channel dimension, height, and width of the feature map, respectively, the network first applies lightweight spatial enhancement to the input features, then flattens them into a sequential format and adds positional information. Subsequently, sequential modeling is performed by the Mamba module. Finally, the spatial structure is restored and combined with the original input via a residual connection. Specifically, the forward pass of this layer comprises the following four steps.

**Spatial Feature Enhancement:** As shown in Figure 1b, the input features are first processed by a lightweight spatial enhancement module. This module employs a depthwise separable convolution design, initially extracting spatial features via a 3×3 grouped convolution (with a group size of C/4), followed by a GELU activation function and a 1×1 convolution for channel mixing. The enhanced features are added to the original features with a weight of 0.1, thereby strengthening small-object features without introducing significant computational overhead. This process is as shown in Formula (9).

(9) $$F_{\mathrm{spatial}}\left(X\right)={\mathrm{Conv}}_{1\times 1}\left(\mathrm{GELU}\left({\mathrm{DepthwiseConv}}_{3\times 3}\left(X\right)\right)\right)$$

**Sequentialization and Positional Encoding:** As shown in Figure 1c, to adapt the two-dimensional visual feature maps to the input requirements of the one-dimensional selective state space model (Mamba) while preserving crucial spatial structural information, this module performs a transformation from the spatial domain to the sequential domain and injects a learnable positional prior. First, the two-dimensional feature map is transformed into a token sequence via a spatial flattening operation:

(10) $$X_{\mathrm{seq}}=Flatten\left(X\right)\in R^{B\times L\times C}$$

where L=H×W is the sequence length. The Flatten(·) operation is specifically defined as $X_{\mathrm{seq}}=reshape{\left(X,\left(B,C,L\right)\right)}^{⊤(0,2,1)}$, i.e., first reshaping the tensor and then transposing dimensions to place the channel dimension *C* last, forming the standard sequence format (B,L,C).

To retain spatial layout awareness after sequentialization, we introduce a compact learnable positional encoding, denoted as $E_{\mathrm{pos}}$. This encoding is implemented as a static, trainable parameter tensor $E_{\mathrm{pos}}\in R^{1\times 1\times C}$. These parameters are initialized as a zero matrix and adaptively learn spatial positional information during training. In the forward pass, the positional encoding is broadcast-added to the flattened sequence:

(11) $$X_{\mathrm{pos}}=X_{\mathrm{seq}}+E_{\mathrm{pos}}$$

Formally, for every batch index *b* and token index *l*,

(12) $$X_{\mathrm{pos}}\left[b,l,:\right]=X_{\mathrm{seq}}\left[b,l,:\right]+E_{\mathrm{pos}}\left[0,0,:\right]$$

This operation effectively assigns a channel-wise, learnable positional bias to every spatial token in the sequence.

The proposed design offers three principal benefits: (1) Parameter efficiency: Only *C* parameters are introduced, irrespective of spatial resolution (H,W), minimizing memory and computational overhead. (2) Adaptive flexibility: It circumvents predefined positional schemes (e.g., sinusoidal functions), enabling the model to learn tailored positional embeddings directly from data. (3) Implementation simplicity: The design avoids complex coordinate computations or interpolations, ensuring ease of integration and stable optimization.

The resulting position-augmented sequence $X_{\mathrm{pos}}$ is then passed to the Mamba state space module for sequential feature modeling.

**State Space Modeling:** The sequentialized features undergo layer normalization before being fed into the Mamba module for state space modeling. This module utilizes a selective state space mechanism, implementing efficient modeling of long-range dependencies through a convolutional scan operation and a discretization process. This process is as shown in Formula (13).

(13) $$X_{\mathrm{mamba}}=Mamba\left(\mathrm{LayerNorm}\left(X_{\mathrm{pos}}\right)\right)$$

**Feature Reconstruction and Residual Connection:** The sequential features output by the Mamba module are reshaped back to the original spatial dimensions B×C×H×W. They are then added to the original input features via a learnable residual weight α (initialized to 0.1) to produce the final output. This process is as shown in Formula (14).

(14) $$X_{\mathrm{out}}=X+\alpha \cdot X_{mamba}$$

#### 3.2.2. Core Component Design

The innovations of this module are threefold: (1) It combines a selective state space model with convolutional operations, balancing global dependency modeling and local feature extraction. (2) Its lightweight design achieves a balance between parameter efficiency and performance. (3) The learnable residual mechanism enhances training stability. In the subsequent experimental section, we will validate the effectiveness of each component design through ablation studies.

**Lightweight Spatial Enhancement Module:** To address small-object detection tasks, we designed a parameter-efficient spatial enhancement module. This module adopts a two-stage structure: the first stage uses grouped depthwise convolution (with a group size of C/4 to extract local spatial features, while the second stage employs pointwise convolution for channel information fusion. This design maintains spatial awareness while significantly reducing the number of parameters (by approximately 75%).

**Selective State Space Model:** The sequentialized features are first normalized and then modeled by the selective state space mechanism of the Mamba module. The Mamba module employs a selective state space mechanism, enabling dynamic focus on important features through an input-dependent selective scanning process. Compared to traditional attention models, this mechanism offers linear time complexity and can efficiently process long-sequence visual features.

**Learnable Residual Mechanism:** A learnable residual weight parameter α is introduced, allowing the network to adaptively adjust the fusion ratio between the state-space-modeled features and the original features. This parameter is initialized to a small value, facilitating stable training, and is adjusted during the optimization process.

It should be noted that this module can be flexibly integrated into existing convolutional neural networks as a feature enhancement layer. Experiments demonstrate that the module effectively improves model performance across various vision tasks, notably achieving significant average precision gains in small-object detection.

### 3.3. PEMBottleneck Module

**Figure 2:**
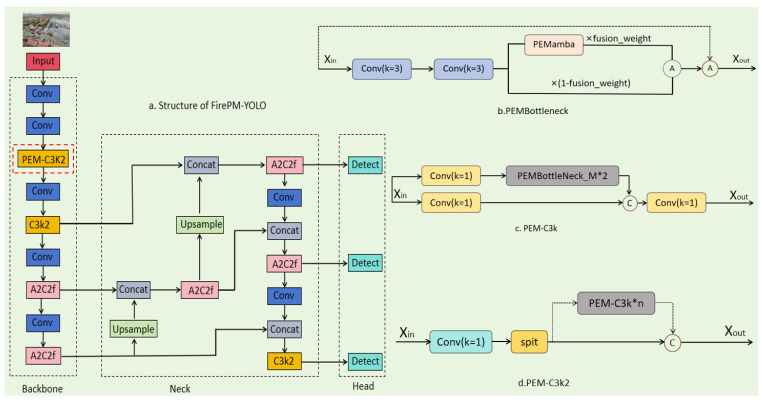
Architecture of the proposed FirePM-YOLO and its core components. (**a**) Overall structure of FirePM-YOLO, illustrating the backbone, neck, and head. The red dashed box highlights the modified part: the first C3K2 module in the backbone is replaced with our proposed PEM-C3K2 module. All other components remain identical to the baseline YOLOv12. (**b**) Detailed structure of the PEMBottleneck module, which consists of parallel convolutional and PEMamba paths fused via a learnable weight. (**c**) Structure of the PEM-C3K module, which serves as the basic building block of PEM-C3K2. (**d**) Structure of the proposed PEM-C3K2 module, composed of multiple serial PEM-C3K modules, enabling hybrid local-global feature modeling at the shallow stage of the backbone.

**Figure 3:**
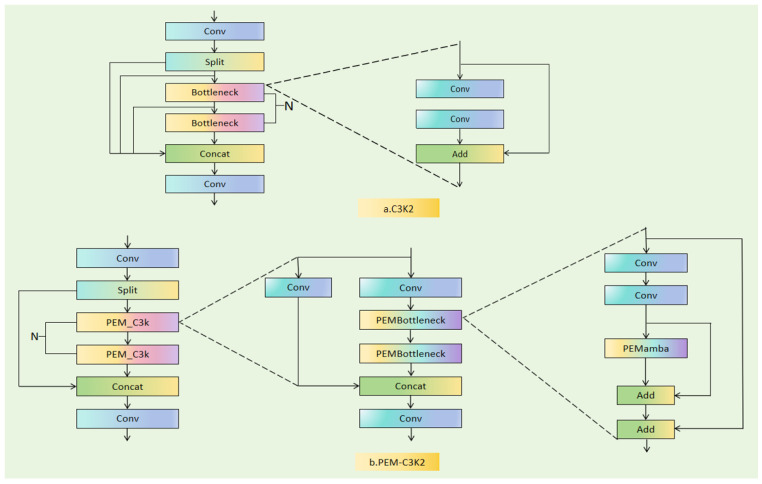
Comparison of the structural diagrams of PEM-C3K2 and standard C3K2. (**a**) Structure of the standard C3K2 module in YOLOv12, composed of multiple serial standard bottlenecks. Each standard bottleneck contains two convolutional branches fused via element-wise addition, limiting the module to local feature extraction. (**b**) Structure of the proposed PEM-C3K2 module, where each standard bottleneck is replaced with a PEM-C3K module. Each PEM-C3K module is built upon PEMBottleneck blocks, enabling parallel local-global feature modeling with learnable fusion weights. This side-by-side comparison highlights the core architectural innovation: transforming purely convolutional feature extraction into hybrid local-global modeling at the shallow stage of the backbone.

In fire rescue scenarios, critical targets such as firefighting vehicles and rescue personnel often exhibit the following characteristics: spatially widespread distribution (targets occupy only a small pixel area from a UAV), significant long-range dependencies (e.g., smoke diffusion and flame spread), and severe local occlusion (in scenarios such as structural collapses, equipment accumulation, or fire trucks being blocked by other objects, which often lead to partial visibility of targets). Traditional convolutional bottleneck structures, due to their limited receptive fields and static computational graphs, struggle to effectively model these complex patterns. To address this, this paper proposes the PEMBottleneck module (see Figure 2b or Figure 3b), which integrates our designed PEMamba module in parallel with convolutional operations, thereby enhancing global dependency modeling while preserving local detail features, achieving a more balanced integration of local information and global context.

The detailed computational procedure of the proposed module is described below. The module accepts input features $X\in R^{C_{in}\times H\times W}$ and produces output features $Y\in R^{C_{out}\times H\times W}$. Its core computational process can be formally expressed as

(15) $$Y=\left\{\begin{array}{cc} X+F_{\mathrm{fused}2}\left(X\right), & \mathrm{if}\;C_{\mathrm{in}}=C_{\mathrm{out}}\;\mathrm{and}\;\mathrm{shortcut}\;\mathrm{enabled}\\ F_{\mathrm{fused}2}\left(X\right), & \mathrm{otherwise} \end{array}\right.$$

where $F_{fused2}$ denotes the fusion function, which will be elaborated in detail in the subsequent section.

The module consists of three core components:

**Local Feature Path:** This path follows the standard bottleneck design, extracting local spatial features through a two-stage convolution, as formalized in Equation (16).

(16) $$\;F_{\mathrm{conv}}={\mathrm{Conv}}_{k_{2}}\left({\mathrm{Conv}}_{k_{1}}\left(X\right)\right)$$

where $Conv_{k_{1}}$ denotes a 3×3 convolutional layer that maps the input space $R^{C_{\mathrm{in}}\times H\times W}$ to $R^{C_{mid}\times H\times W}$, thereby reducing the channel dimensionality. $Conv_{k_{2}}$ is the 3×3 grouped convolutional layer (with group count g=1) mapping $R^{C_{mid}\times H\times W}$ to $R^{C_{\mathrm{out}}\times H\times W}$.

**Global Context Path:** To capture long-range spatial dependencies, the module incorporates a pemamba layer (Equation (17)).

(17) $$\;F_{\mathrm{pemamba}}=\mathrm{PEMamba}\left(F_{\mathrm{conv}};d_{\mathrm{state}},E,L\right)$$

where pemamba denotes the pemambaMambaLayermodule, configured with a hidden state dimension $d_{\mathrm{state}}$, an expansion factor E=4 and L=2 successive SSM layers. The pemamba module dynamically models global feature dependencies via a selective scanning mechanism. Its discrete state-space equations are given in Equations (5) and (6).

**Adaptive Fusion:** To balance local details with global contextual information, the module employs a learnable weighted fusion mechanism (Equation (18)).

(18) $$\;F_{\mathrm{fused}2}=\left(1-\beta \right)F_{\mathrm{conv}}+\beta F_{\mathrm{pemamba}}$$

where β denotes a learnable fusion weight, initialized to 0.3 and optimized via gradient descent during training. If the pemamba path is disabled (use_pemamba = False), the module degenerates to a standard bottleneck structure, ensuring flexibility and extensibility of the architecture.

To facilitate subsequent architecture search and computational optimization, the module incorporates a configurable switch use_pemamba. When set to False, it reduces to the standard bottleneck structure:

(19) $$F_{\mathrm{fused}2}=\left\{\begin{array}{cc} \left(1-\beta \right)F_{\mathrm{conv}}+\beta F_{\mathrm{pemamba}}, & \mathrm{if}\;use\_pemamba=True\\ F_{\mathrm{conv}}, & \mathrm{otherwise} \end{array}\right.$$

Compared to the standard convolutional bottleneck, the PEMamabaBottleneck offers the following theoretical advantages:

1. **Receptive Field Expansion:** The pemamba path provides a global receptive field, compensating for the locality limitations of convolutional operations.
2. **Dynamic Modeling Capability:** The selective mechanism enables the model to focus on spatial regions relevant to firefighting and rescue tasks, such as the direction of flame spread and connected smoke regions.
3. **Computational Efficiency:** Mamba’s linear complexity $O(L\cdot d_{\mathrm{state}}^{2})$ ensures that global modeling does not introduce excessive computational overhead.
4. **Interpretability:** The value of the fusion weight β can reflect the network’s degree of reliance on global context.

**Rationale for Parallel Fusion Architecture.** A critical design choice in PEMBottleneck is the parallel fusion of convolutional features and PEMamba features, rather than a cascaded or serial integration. This decision is grounded in the complementary nature of these two feature representations:

**(1) Complementary Feature Characteristics:** Convolutional operations excel at extracting local patterns—edges, textures, and fine-grained details—through their inductive bias of local connectivity and weight sharing. However, they struggle with long-range dependencies due to limited receptive fields. Conversely, PEMamba captures global contextual relationships through its selective state space mechanism but may sacrifice some local precision. A parallel architecture allows both pathways to operate simultaneously on the same input, preserving the distinct strengths of each representation without forcing one to pass through the other, which could cause information loss or distortion.

**(2) Gradient Flow Optimization:** In serial architectures where one module feeds into the other, gradients must propagate through both pathways sequentially, potentially leading to vanishing gradients or optimization difficulties. Our parallel design with learnable fusion weights creates two independent gradient highways—gradients can flow directly to either pathway, facilitating more stable training and better convergence. This is particularly important for the newly introduced PEMamba module, which has different optimization dynamics than standard convolutions.

**(3) Adaptive Feature Balancing via Learnable Fusion:** The learnable fusion weight β (initialized to 0.3) enables the network to dynamically adjust the contribution of global context versus local details based on the specific characteristics of each spatial location and each sample. For regions with clear local patterns (e.g., visible vehicle edges), the network may rely more on convolutional features; for ambiguous regions requiring contextual disambiguation (e.g., smoke-obscured areas), it can upweight PEMamba features. This spatial-adaptive fusion capability would be impossible in serial designs where the weighting is implicit and fixed by the network depth.

**(4) Ablation Validation:** As evidenced in Table 3, the parallel fusion design (configuration “B + C”) achieves superior performance compared to both the baseline and configurations without fusion, with gains of +3.90% in mAP50 and +5.76% in mAP50-95. Notably, these improvements come without increasing parameter count, confirming that the performance gain stems from the architectural synergy rather than model capacity expansion.

### 3.4. The FirePM-YOLO Architecture

#### 3.4.1. YOLOv12 Baseline Architecture

Before describing our proposed architecture, we briefly review the structure of YOLOv12, which serves as our baseline. As illustrated in Figure 2a (excluding the red dashed box), YOLOv12 follows a standard backbone–neck–head design:

**Backbone:** A series of convolutional stages for feature extraction. The backbone contains multiple C3K2 modules at different scales. The first C3K2 module (see Figure 3a) operates on high-resolution feature maps (160 × 160 for 640 × 640 inputs), capturing fine-grained spatial details essential for small object detection. Subsequent C3K2 modules operate on progressively downsampled feature maps (80 × 80, 40 × 40, 20 × 20), trading spatial resolution for semantic richness.

**Neck:** A feature pyramid network that aggregates multi-scale features from different backbone stages, enabling detection across varied object sizes.

**Head:** The detection head that predicts bounding boxes and class probabilities from the neck’s features.

Each C3K2 module in YOLOv12 consists of multiple serial standard bottlenecks (see Figure 3a), with each bottleneck containing two convolutional branches fused via element-wise addition. This design, while computationally efficient, is inherently limited to local feature extraction—each convolution operates within a fixed kernel window, and receptive fields grow only linearly with depth.

#### 3.4.2. FirePM-YOLO: Integrating PEMamba into YOLOv12

To address the limitations of YOLOv12 in capturing global context—particularly critical for fire rescue scenarios with large-scale smoke patterns and occluded targets—we propose FirePM-YOLO (see Figure 2a). As highlighted by the red dashed box in Figure 2a, our modification is highly localized and intentional: we replace only the first C3K2 module in the backbone with our proposed PEM-C3K2 (see Figure 2d or Figure 3b). All other components—including the remaining backbone stages, the neck, and the head—are identical to YOLOv12.

This minimal modification design serves two purposes: (1) it allows us to isolate and evaluate the contribution of the PEMamba module without confounding factors from architectural changes elsewhere, and (2) it preserves YOLOv12’s proven efficiency in all other components.

#### 3.4.3. PEM-C3K2 Module

While Section 3.2 and Section 3.3 have detailed the internal workings of PEMamba and PEMBottleneck, here we describe how these components come together to form PEM-C3K2. As shown in Figure 3b, PEM-C3K2 maintains the same overall structure as standard C3K2—it consists of multiple serial blocks. However, each standard bottleneck is replaced with a PEMBottleneck (Section 3.3). The relationship is straightforward:

Standard C3K2 = serial [Bottleneck, Bottleneck, …]

PEM-C3K2 = serial [PEMBottleneck, PEMBottleneck, …]

Within each PEMBottleneck, the parallel paths (convolutional and PEMamba) operate independently and are fused via learnable weight β, as detailed in Section 3.3. Figure 3a,b provide a side-by-side visual comparison of these two module types.

#### 3.4.4. Differences Between PEM-C3K2 and Standard C3K2

Figure 3 provides a side-by-side comparison of the standard C3K2 module and our proposed PEM-C3K2 module. The core differences between these two modules are manifested in the following three aspects:

**(1) Compositional Structure.** The standard C3K2 consists of multiple serial standard bottlenecks. Each bottleneck contains only two convolutional branches, with features fused through element-wise addition. In contrast, the PEM-C3K2 replaces each standard bottleneck with our proposed PEMBottleneck. Each PEMBottleneck contains two parallel paths: a convolutional path responsible for extracting fine-grained local details and a PEMamba path responsible for capturing global contextual features. These two paths are fused via a learnable weight, enabling adaptive balancing of local and global information.

**(2) Receptive Field Characteristics.** The receptive field of the standard C3K2 grows linearly with network depth, requiring the stacking of multiple layers to cover large spatial extents, which limits its capacity for modeling global information. Conversely, the PEM-C3K2 achieves a global receptive field within each PEMBottleneck through the state space mechanism of PEMamba. This allows the network to acquire scene-level contextual awareness at shallow stages, providing early guidance for subsequent feature extraction.

**(3) Feature Representation Capability.** The standard C3K2 extracts features solely through convolutional operations, resulting in a single, homogeneous representation. The PEM-C3K2, however, maintains two complementary representations—convolutional features and state space features—simultaneously. The learnable fusion weight β enables spatially adaptive feature balancing: in regions with clear target boundaries, the network can rely more on convolutional details; in ambiguous areas such as those obscured by smoke, it can depend more on the contextual reasoning provided by PEMamba.

#### 3.4.5. Strategic Placement of PEMamba in Shallow Layers

A key design decision in our architecture is the deliberate placement of the PEMamba module exclusively in the shallow layers of the backbone (specifically, the first C3K2 module). This choice is motivated by the unique characteristics of fire rescue scenarios and the nature of small object detection:

**(1) Resolution Preservation for Small Targets:** In UAV-based fire reconnaissance, critical targets such as firefighters and small rescue vehicles often occupy only a few dozen pixels. Shallow feature maps retain higher spatial resolution, providing richer fine-grained details essential for detecting these small-scale objects. Placing PEMamba in deeper layers would suffer from severe spatial information loss due to successive downsampling, making small target detection inherently difficult regardless of subsequent feature enhancement.

**(2) Early Context Integration:** Fire rescue scenes are characterized by large-scale contextual patterns—smoke plumes, flame spread directions, and structural layouts—that provide crucial cues for locating partially occluded or ambiguous targets. By integrating global context modeling at shallow stages, PEMamba injects this contextual awareness early in the feature extraction pipeline, guiding subsequent convolutional layers to focus on task-relevant regions. This “context-aware early encoding” strategy contrasts with approaches that only model global dependencies at deep stages, where spatial details have already been compromised.

**(3) Hierarchical Feature Refinement:** Our design follows a “global context priming + local detail refinement” paradigm: PEMamba first establishes broad scene understanding through its selective state space mechanism, then the following convolutional layers iteratively refine local details while being guided by this global context. This hierarchical approach mimics human visual perception in complex scenes—first grasping the overall scene structure, then scrutinizing specific regions of interest.

**(4) Computational Efficiency Trade-off:** Mamba’s linear complexity becomes more computationally demanding as spatial resolution increases. Placing PEMamba at the earliest stage (highest resolution) would incur unacceptable latency for real-time UAV deployment. Our chosen position—after initial downsampling but before severe resolution reduction—strikes an optimal balance between spatial detail preservation and computational efficiency.

## 4. Experimental Results and Analysis

### 4.1. Experimental Environment and Hyperparameter Settings

All experiments in this study were conducted on a Linux operating system, with detailed specifications provided in Table 1. Model training and validation were conducted based on the Ultralytics YOLO framework. The main hyperparameter settings used are shown in Table 2.

### 4.2. Experimental Dataset

**Figure 4:**
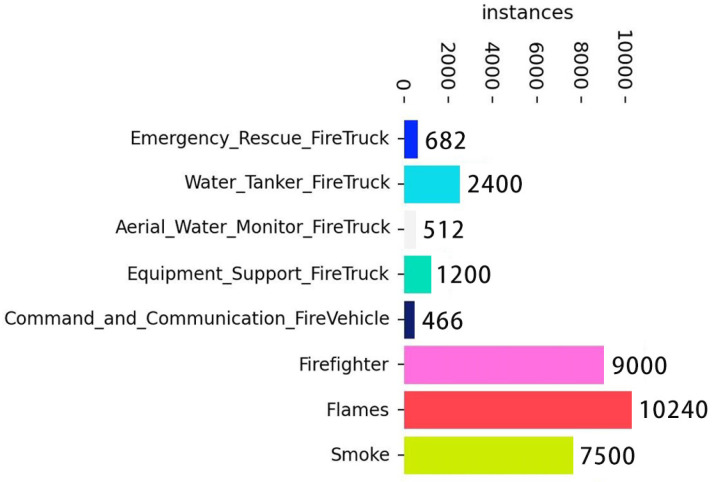
Annotation statistics of the FireRescue dataset. This figure shows the distribution of 32,000 annotations across eight categories: Flames (32.00%, 10,240 instances), Firefighter (28.13%, 9000 instances), Smoke (23.44%, 7500 instances), Water Tanker FireTruck (7.50%, 2400 instances), Equipment Support FireTruck (3.75%, 1200 instances), Emergency Rescue FireTruck (2.13%, 682 instances), Command and Communication FireVehicle (1.46%, 466 instances), and Aerial Water Monitor FireTruck (1.60%, 512 instances).

The FireRescue dataset [6] is the first large-scale object detection dataset specifically designed for comprehensive firefighting and rescue scenarios. It comprises 15,980 images with 32,000 high-quality annotations across eight target categories: five types of common fire rescue vehicles, firefighters, smoke, and flames. On average, each image contains approximately 2.00 annotated targets. The detailed category names and instance count distribution are presented in Figure 4. The dataset covers diverse scenes, including urban roads, suburban environments, and night scenes under various weather conditions, with numerous small and occluded targets. These characteristics make the FireRescue dataset particularly suitable for research tasks such as small object detection and robustness evaluation in complex scenes.

Due to the diverse data sources—including real-world fire rescue operations, training exercises, and online repositories—the original images exhibit varying spatial resolutions, typically ranging from 640 × 480 to 1920 × 1080 pixels. To ensure consistent model training, all images are uniformly resized to 640 × 640 pixels during preprocessing, following the standard input requirements of the YOLO series. Aspect ratio is preserved through padding to maintain the original content’s integrity. Finally, the dataset is randomly split into training, validation, and test sets with a ratio of 8:1:1 to ensure fairness and reproducibility in experimental evaluations.

### 4.3. Ablation Study

To validate the effectiveness of the individual improvements proposed in this paper for the enhanced fire rescue scene detection method, a comprehensive ablation study was conducted. All experiments were strictly controlled to ensure consistency in model parameters. The results of the ablation study are presented in Table 3, where “+A” indicates the incorporation of the Mamba improvement, “+B” indicates the incorporation of the PEMamba improvement, and “+C” indicates that a weighted fusion mechanism is adopted in the bottleneck block.

As evidenced in Table 3, the effectiveness of each proposed improvement is validated. The introduction of the Mamba module enhances cross-dimensional information interaction with a negligible increase in computational cost, resulting in gains of +2.93% in mAP50, +4.79% in mAP50-95, +8.02% in precision, and +3.91% in recall, thereby validating its contribution to detection performance. The proposed PEMamba module delivers significant performance gains—+2.94% mAP50, +5.62% mAP50-95, +8.07% precision, and +3.92% recall—while requiring almost no increase in parameters or computational cost. Finally, this final modification yields a further increase of +3.90% in mAP50, +5.76% in mAP50-95, +8.14% in precision, and +5.80% in recall, confirming its substantial benefit to overall model performance.

As shown in Table 3, the number of parameters in the improved models does not increase, indicating that the gains come primarily from structural optimizations rather than computational expansion. In terms of inference speed, FPS (frames per second), all models exhibit varying degrees of decrease: B + C drops the most (−18.9%), followed by B (−18.6%), while A shows the smallest reduction (−16.9%). Although the proposed models experience a decline in inference speed, their FPS during real-time image analysis remains above 30, which fully meets the practical requirements for real-time detection tasks [43].

### 4.4. Comparative Experiments

In this study, we enhance YOLOv12 by integrating novelly designed modules. To comprehensively evaluate the effectiveness of our proposed method, we conduct two sets of comparative experiments based on model scale. First, the proposed module is incorporated into two different scales of YOLOv12, namely YOLOv12n and YOLOv12s, resulting in two new architectures: FirePM-YOLOn and FirePM-YOLOs. Subsequently, a rigorous performance comparison is carried out. The decision to employ both nano (n)- and small (s)-scale models for evaluation is twofold. First, it aims to demonstrate the generalizability and scalability of our proposed module. A common pitfall in architectural innovation is that improvements may be effective only at a specific model capacity. By validating our module across two distinct scales, we rigorously show that its benefits are not merely incidental to a particular parameter count but are a consistent and transferable enhancement. Second, this approach provides a more comprehensive performance characterization. The nanoscale models, with their extreme efficiency, are critical for applications in resource-constrained environments. Improvements here are measured by the gain in performance per parameter. Conversely, the small-scale models offer a higher baseline performance, allowing us to evaluate whether our module can provide a significant boost even when the base architecture is already more powerful. This dual-level assessment offers complete evidence of our module’s practical utility across different performance-efficiency trade-offs.

The detailed performance comparison results of different models are shown in Table 4, from which the following conclusions can be drawn:

The benefits of our module manifest differently across model scales, highlighting its versatile utility. For the computation-bound nanoscale models, the primary advantage lies in a remarkable +3.90% increase in mAP50, +5.76% increase in mAP50-95, +8.14% increase in precision, and +5.80% increase in recall, crucial for reducing missed detections in resource-constrained deployments. Conversely, for the more powerful small-scale models, our module pushes the performance ceiling further, achieving a significant +0.73% increase in mAP50, +1.64% increase in mAP50-95, +0.31% increase in precision, and +0.33% increase in recall, which is vital for high-accuracy applications.

Crucially, our enhanced model FirePM-YOLOn outperforms the original YOLOv12n by a clear margin, while both models possess an identical number of parameters. This provides direct and fair evidence that the improvement stems from our architectural innovation, not simply an increase in model size. The same trend holds for the small-scale group, where FirePM-YOLOs consistently surpasses YOLOv12s.

Through comprehensive analysis, our final model, FirePM-YOLO, achieves the optimal balance in overall performance among all compared models.

**Per-Category Performance Analysis.** To comprehensively evaluate the effectiveness of the proposed method, Table 5 presents the per-category AP50 comparison between YOLOv12n and FirePM-YOLO across all eight categories in the FireRescue dataset. The results demonstrate that FirePM-YOLO achieves performance improvements in six out of eight categories, maintains parity in one category, and shows only a marginal decrease in a single category, confirming the stability and generalization capability of the proposed approach. Notably, the most substantial gain (+0.08, from 0.51 to 0.59, representing a relative improvement of 15.7%) is observed in the Emergency Rescue FireTruck category—the class with the lowest baseline performance—validating that the PEMamba module’s spatial position awareness is particularly effective for challenging, hard-to-detect targets. Steady improvements are also achieved on the Aerial Water Monitor FireTruck (+0.06, 9.1% relative gain) and the Water Tanker FireTruck (+0.04, 5.6% relative gain). For the non-vehicle categories of particular reviewer interest, smoke detection improves from 0.78 to 0.80 (+0.02, 2.6% relative gain), while flame detection increases from 0.69 to 0.70 (+0.01, 1.4% relative gain). These gains, achieved on top of already competitive baselines, demonstrate that global context modeling benefits even categories with amorphous boundaries and dynamic appearances such as smoke and flames. Firefighter detection shows a slight increase from 0.34 to 0.35 (+0.01, 2.9% relative gain), reflecting the inherent difficulty of detecting small human targets in complex fire scenes. Collectively, these results validate that FirePM-YOLO achieves a practical performance balance—delivering significant improvements on core rescue vehicle detection (notably the 15.7% relative gain on Emergency Rescue FireTruck) while maintaining competitive performance on environmental perception categories—aligning with the real-world requirements of fire rescue operations.

For the non-vehicle categories of the FireRescue dataset, smoke detection improves from 0.78 to 0.80 (+0.02, 2.6% relative gain), while flame detection increases from 0.69 to 0.70 (+0.01, 1.4% relative gain). Although these gains are modest in absolute terms, achieving further improvement on top of already competitive baselines demonstrates that global context modeling benefits even categories with amorphous boundaries and dynamic appearances such as smoke and flames. Firefighter detection shows a slight increase from 0.34 to 0.35 (+0.01, 2.9% relative gain), reflecting the inherent difficulty of detecting small human targets in complex fire scenes.

Collectively, these results validate that FirePM-YOLO achieves a practical performance balance—delivering significant improvements on core rescue vehicle detection (notably the 15.7% relative gain on Emergency Rescue FireTruck) while maintaining competitive performance on environmental perception categories—aligning with the real-world requirements of fire rescue operations.

### 4.5. Visualization of Experimental Results

To provide an intuitive comparison of the detection performance between the proposed improved model and the baseline model, challenging images were selected from the test set for comparative evaluation. The superiority of the improved model is demonstrated through two visualization methods: detection results and detection heatmaps.

#### 4.5.1. Visualization of Detection Results

This subsection qualitatively demonstrates the performance improvement of our proposed FirePM-YOLO across three challenging scenarios through comparative experiments. For a comprehensive evaluation, we compare the “n” scale models in the first and third experimental groups to assess their performance under lightweight configurations, while the “s” scale models are used in the second group to investigate their detection capability with higher computational budgets.

(1) Small and Occluded Object Detection Capability

**Figure 5:**
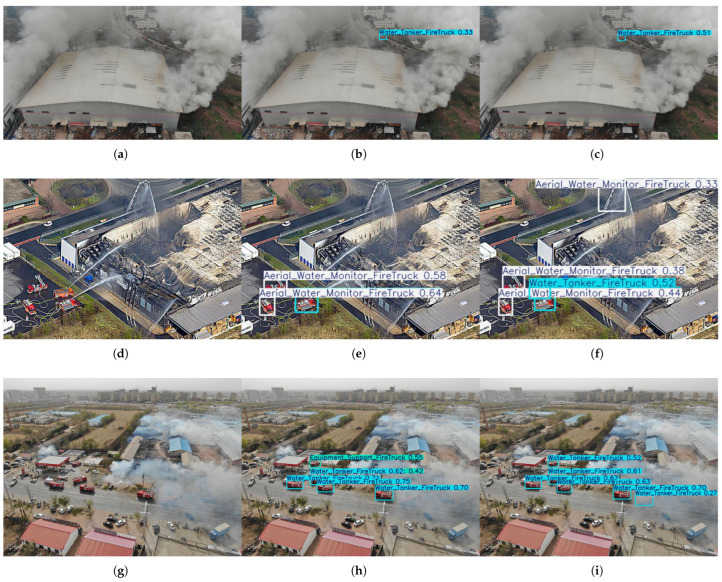
Comparison of detection results between YOLOv12n and FirePM-YOLO under small and occluded object detection scenarios. (**a**–**c**) Experimental results on long-distance ultra-small targets. (**d**–**f**) Experimental results on heavy occlusion of small targets by building debris. (**g**–**i**) Experimental results on soft occlusion of small targets by smoke.

**Case 1: Long-distance ultra-small target.** As shown in Figure 5a–c, this scenario captures a real firefighting and rescue scene from the high-altitude reconnaissance perspective of a UAV. The fire truck, as the core detection target, only occupies approximately 30 × 50 pixels in the image, which is a typical long-distance small target in actual fire rescue scenarios. The comparative detection results show that the baseline model YOLOv12n achieves a detection confidence of merely 0.33 for the fire truck target (Figure 5b), while the proposed FirePM-YOLO model in this paper raises the detection confidence for the same target to 0.51 (Figure 5c). This result fully verifies that the spatial position enhancement mechanism designed in the PEMamba module can effectively strengthen the feature representation of small targets and preserve key spatial information, thus significantly improving the detection confidence and recognition performance of long-distance small targets in real firefighting and rescue scenes from the UAV’s high-altitude perspective.

**Case 2: Heavy occlusion of small targets by building debris.** As shown in Figure 5d–f, the first experiment was designed to evaluate the model’s detection capability for small and partially occluded objects from a drone perspective. The experimental scenario is based on a real fire scene with complex backgrounds: due to the long observation distance, multiple fire trucks occupy only minimal pixel areas in the image, while some vehicles are partially occluded by surrounding objects. Under these highly challenging conditions, the baseline YOLOv12n model exhibited noticeable missed detections. In contrast, our proposed FirePM-YOLOn model demonstrated significantly improved detection performance: it not only successfully detected all visible fire vehicles missed by the baseline model but also accurately identified a specialized aerial ladder fire truck by leveraging its unique folding arm structure—a key local feature entirely overlooked by the baseline model. This ability to detect and classify specialized equipment based on local structural features directly validates the effectiveness of the global contextual awareness mechanism and the multi-scale feature enhancement method employed in our model.

**Case 3: Soft occlusion of small targets by smoke.** As shown in Figure 5g–i, the experimental scenario is an aerial reconnaissance scene at a gas station. A fire truck is performing fire-extinguishing operations in a dense smoke area, and its body is softly occluded by white smoke, resulting in extremely low contrast between the target and the background. The YOLOv12n model fails to detect this target, while FirePM-YOLOn successfully detects it with a confidence score of 0.27. The lightweight spatial enhancement module in PEMamba effectively improves the contrast between small targets and the smoke background, suppresses the noise interference caused by dynamic smoke textures, and thus achieves stable detection of small targets under soft occlusion.

(2) Detection Robustness under Extreme Visual Interference

**Figure 6:**
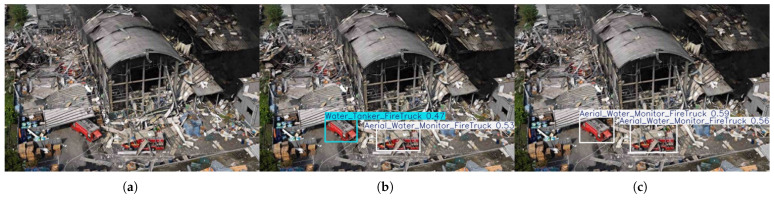
Comparison of detection results between YOLOv12n and FirePM-YOLO under extreme visual interference. (**a**) Original image featuring a fire truck partially buried under collapsed building debris, representing severe occlusion and fragmented target morphology. (**b**) Detection results of YOLOv12n, showing low confidence (0.47 and 0.53) and imprecise localization. (**c**) Detection results of FirePM-YOLO, achieving high confidence (0.59 and 0.56) and accurate localization through global context modeling.

A second comparative experiment was designed to evaluate model robustness under extreme visual interference, particularly in scenarios involving severe target occlusion. As depicted in Figure 6a–c, the test scenario simulates a disaster scene where a collapsing, burning building has partially buried a fire truck under structural debris. This setup introduces significant visual challenges: the target suffers severe occlusion from irregular, large-scale solid rubble, resulting in fragmented geometric morphology and substantial loss of surface texture information. Experimental results demonstrate that our proposed FirePM-YOLO model exhibits superior detection robustness and confidence stability compared to the baseline YOLOv12 under such extreme occlusion conditions. As shown in Figure 6b, the baseline model shows notable deficiencies in detecting the buried fire truck—a critical rescue target. While capable of coarse localization, it produces low confidence scores of 0.47 and 0.53 for the two visible instances, with noticeable imprecision in bounding box localization. Such low-confidence detections are particularly vulnerable to filtering by confidence thresholds in practical deployment, potentially leading to missed detections and compromising rescue decision-making. In contrast, Figure 6c illustrates that our FirePM-YOLO model achieves higher confidence scores of 0.59 and 0.56 for the same occluded fire trucks under identical conditions, along with more precise localization. This enhancement directly validates the effectiveness of our model’s feature extraction and fusion mechanisms, which enable superior integration of visible local features (e.g., vehicle color, wheels, partially exposed chassis) with contextual information (e.g., fire environment, debris distribution, typical vehicle positioning). The PEMamba module’s global context modeling capability allows the network to infer the presence of targets even when local visual evidence is partially compromised by leveraging surrounding scene cues.

(3) Multi-Target Discrimination Capability in Dense Scenes

**Figure 7:**
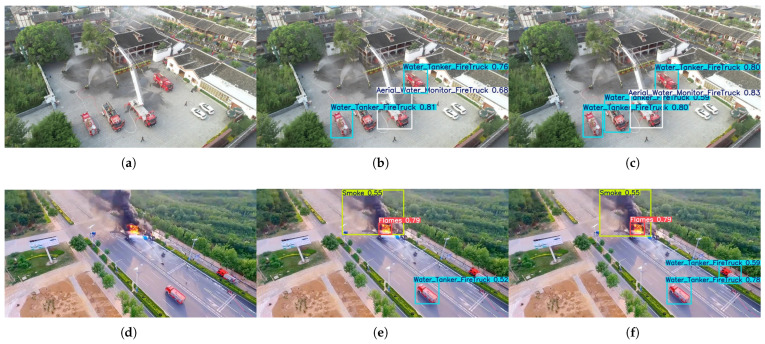
Comparison of detection results between YOLOv12n and FirePM-YOLO in multi-target discrimination scenarios. First row (**a**–**c**): Dense multi-vehicle scenario. (**a**) Original image featuring multiple types of fire vehicles densely distributed. (**b**) Detection results of YOLOv12n. (**c**) Detection results of FirePM-YOLO. Second row (**d**–**f**): Complex environment with smoke and flame interference. (**d**) Original image where fire vehicles coexist with smoke and flames. (**e**) Detection results of YOLOv12n. (**f**) Detection results of FirePM-YOLO.

This set of experiments aims to evaluate the detection performance of the model in scenarios with densely coexisting multi-type fire trucks and complex environmental interference. As shown in Figure 7a–c, in scenes with densely distributed fire trucks such as ladder trucks and water tankers, the baseline model YOLOv12n suffers from obvious missed detections and fails to identify all targets completely. In contrast, the proposed FirePM-YOLOn model exhibits significantly improved multi-target discrimination capability: it not only successfully detects all visible fire trucks but also accurately distinguishes different types of fire vehicles without category confusion or target omission. These results validate the ability of the proposed method to maintain feature discriminability in dense scenes. Furthermore, Figure 7d–f presents the detection results in complex environments where fire trucks, smoke, and flames coexist. In terms of smoke and flame detection, YOLOv12n and FirePM-YOLOn achieve similar confidence scores, indicating that the two models have comparable perception ability for such non-rigid targets. However, for the core target, i.e., fire trucks, YOLOv12n still shows obvious missed detections, while FirePM-YOLOn successfully identifies all fire vehicles. This comparison further verifies the superiority of the proposed model in detecting fire trucks as key targets. It demonstrates that the global context modeling ability of the PEMamba module can effectively overcome smoke interference and enhance the detection robustness for rigid small targets.

#### 4.5.2. Heatmap Visualization and Analysis

**Figure 8:**
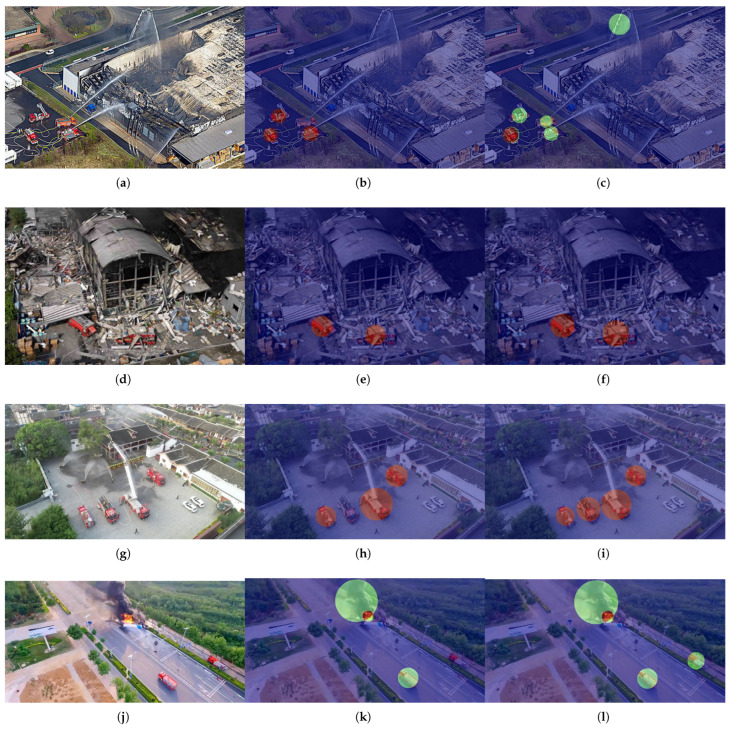
Comparative visualization of the heatmap results between YOLOv12n and FirePM-YOLO. (**a**,**d**,**g**,**j**) are the original images; (**b**,**e**,**h**,**k**) show the heatmap visualization results of YOLOv12n; and (**c**,**f**,**i**,**l**) present the heatmap visualization results of FirePM-YOLO.

To gain deeper insight into the models’ internal decision-making processes and to explain the performance differences observed in the detection results, we employ Grad-CAM to generate corresponding heatmaps for the same three sets of scenarios, visualizing the models’ attention regions during inference. Figure 8 presents the heatmap visualizations of both the baseline model and the improved model when processing challenging images from the test set. In the heatmaps, deeper red coloration indicates higher model attention to the corresponding feature regions.

As illustrated in Figure 8, the heatmap visualizations from the four sets of comparative experiments clearly reveal the underlying cause of missed detections in the baseline model. Across all three dense scenarios, the heatmaps of our model consistently demonstrate highly concentrated activation responses toward all critical targets, including partially occluded ones. The model’s attention is sharply focused on discriminative regions of each target, forming clear and localized high-activation peaks. In contrast, the heatmaps of the baseline model directly explain its frequent missed detections: in regions corresponding to missed targets, the model produces almost no significant feature activation. These areas exhibit low-intensity heatmap coloration similar to the background, indicating that these targets failed to obtain effective feature representation during the feature extraction stage. Furthermore, the attention of the baseline model is noticeably scattered—activations are often misplaced onto background textures, irrelevant objects, or the surroundings of already detected prominent targets, rather than being evenly distributed across all potential objects. This comparative visualization provides empirical support for the effectiveness of the multi-scale attention mechanism and feature enhancement modules introduced in our model. These components substantially improve the model’s feature sensitivity and spatial discriminability in dense scenes, ensuring that all potential targets receive sufficient activation in the feature maps and are successfully recalled in subsequent detection stages. These observations also offer an intuitive explanation for the quantitative improvements reported in Table 4.

## 5. Conclusions

This paper proposes FirePM-YOLO, a novel object detection framework designed to address the specific challenges encountered in UAV-based fire rescue scenarios. By integrating the custom-designed Position-Aware Enhanced Mamba (PEMamba) module into the YOLOv12 architecture, the model achieves a significant enhancement in perceiving small-scale targets, handling severe occlusions, and resisting visual interference from smoke and flames, which are common in fireground environments. The core innovation lies in the PEMamba module, which introduces a lightweight spatial enhancement mechanism, learnable positional embeddings, and a selective state space model within a unified layer. This design effectively combines the global contextual modeling capability of Mamba with the local feature extraction strength of convolutional operations. Furthermore, the proposed PEMBottleneck structure, which replaces standard bottlenecks in the shallow layers of the backbone, enables the model to capture long-range dependencies across the scene while preserving fine-grained details crucial for small object detection, all with linear computational complexity. In conclusion, FirePM-YOLO provides an effective and efficient solution for real-time, high-precision object detection in the critical domain of fire rescue. This work not only advances the state-of-the-art for vision-based fireground analysis but also presents a valuable paradigm for integrating state space models into convolutional detection frameworks to tackle classic challenges such as small object detection in complex environments. Future work may involve further optimization for deployment on resource-constrained UAV platforms and extension to multi-modal or temporal analysis for enhanced situational awareness.

## Figures and Tables

**Table 1:** Experimental environment.

Item	Configuration
CPU	Intel(R) Core(TM) i9-14900HX
GPU	NVIDIA GeForce RTX H800
GPU Memory	80 G
Pytorch	Pytorch 2.4.1
Python	Python 3.10.20
CUDA	CUDA 11.8
Utralytics	8.3.170

**Table 2:** Training hyperparameter settings.

Hyperparameter	Value	Description
Epochs	250	Total number of training epochs
Batch Size	96	Number of images processed per batch
Initial Learning Rate	0.01	Starting learning rate for training
Final Learning Rate	0.0001	Final learning rate (lr0 × lrf)
Optimizer	SGD	Automatically select the optimal optimizer
Momentum	0.937	SGD momentum/Adam beta1
Weight Decay	0.0005	Regularization parameter
Data Loading Workers	48	Number of parallel data loading threads

**Table 3:** Ablation study on the FireRescue dataset.

Model	mAP50	mAP50-95	Precision	Recall	Params(M)	GFLOPs	FPS
baseline	0.65869	0.38103	0.79117	0.62561	2.6	6.5	119.48
+A	0.67804	0.39927	0.85465	0.65005	2.6	6.5	99.28
+B	0.67811	0.40243	0.85501	0.65014	2.6	6.5	97.27
B + C	0.68436	0.40297	0.85558	0.66185	2.6	6.5	96.94

**Table 4:** Performance comparison of different models on the FireRescue dataset.

Model	mAP50	mAP50-95	Precision	Recall	Params (M)	GFLOPs	FPS
YOLOv3-tiny [13]	0.61258	0.34372	0.7831	0.58579	2.5	7.1	394.29
YOLOv5nu	0.64649	0.37861	0.7688	0.60921	2.6	7.7	225.61
YOLOv6n [16]	0.63701	0.37049	0.79729	0.5851	4.2	11.7	255.67
YOLOv8n [18]	0.66694	0.39639	0.79785	0.63559	3.5	10.5	240.14
YOLOv9t [19]	0.54452	0.31304	0.76716	0.54023	2.0	7.7	115.91
YOLOv10n [22]	0.66728	0.3971	0.8188	0.63906	2.3	6.7	195.40
YOLOv11n [23]	0.53933	0.31657	0.77228	0.53967	2.6	6.4	188.23
YOLOv12n [2]	0.65869	0.38103	0.79117	0.62561	2.6	6.5	119.48
VMamba-YOLOv12n [41]	0.6693	0.39581	0.79946	0.62633	2.6	6.8	109.84
Mamba-YOLOt [42]	0.66546	0.39783	0.78829	0.62745	6.0	13.6	74.58
FirePM-YOLOn	0.68436	0.40297	0.85558	0.66185	2.6	6.5	96.94
RTDetr [44]	0.55143	0.31893	0.7459	0.56918	320.0	103.5	34.96
YOLOv3 [13]	0.67386	0.4077	0.80284	0.64746	103.6	282.2	132.52
YOLOv5su [16]	0.68456	0.40947	0.78673	0.66119	9.1	24.1	222.47
YOLOv6s [16]	0.65418	0.38075	0.79444	0.61829	16.3	43.9	245.10
YOLOv8s [18]	0.68854	0.36942	0.79609	0.64973	11.4	29.7	235.54
YOLOv9s [19]	0.69309	0.41547	0.7823	0.665	7.2	26.7	109.04
YOLOv10s [22]	0.68024	0.40995	0.78448	0.6569	8.0	24.8	185.56
YOLOv11s [23]	0.68558	0.40973	0.81349	0.66232	9.4	21.5	186.40
YOLOv12s [2]	0.69381	0.41192	0.7852	0.6741	9.2	21.2	116.71
RGM-YOLO [27]	0.68879	0.40537	0.7796	0.6645	7.2	26.7	109.04
YOLOv7scb [26]	0.66879	0.38723	0.79714	0.63266	33.39	171.5	125.48
Mamba-YOLOb [42]	0.68423	0.40529	**0.81729**	0.64268	21.8	49.6	74.81
FirePM-YOLOs	0.69889	0.41868	0.78763	0.67632	9.2	21.8	94.85

**Table 5:** Per-category AP50 comparison on the FireRescue dataset.

Model	Emergency Rescue FireTruck	Water Tanker FireTruck	Aerial Water Monitor FireTruck	Equipment Support FireTruck	Communication FireVehicle	Firefighter	Flames	Smoke
YOLOv12n	0.51	0.72	0.66	0.78	0.73	0.34	0.69	0.78
FirePM-YOLOn	0.59	0.76	0.72	0.77	0.73	0.35	0.70	0.80
Improvement	+0.08	+0.04	+0.06	−0.01	0.00	+0.01	+0.01	+0.02

## Data Availability

Data are contained within the article.
